# Intraoperative cell salvage as an effective intervention for postpartum hemorrhage—Evidence from a prospective randomized controlled trial

**DOI:** 10.3389/fimmu.2022.953334

**Published:** 2022-10-10

**Authors:** Bo Lei, Min Guo, Xin Deng, Shujun He, Xin Lu, Yunjuan Wang, Lei Wang

**Affiliations:** ^1^ Department of Anesthesiology, Haidian Maternal and Child Health Hospital, Beijing, China; ^2^ Clinical Laboratory, Haidian Maternal and Child Health Hospital, Beijing, China

**Keywords:** intraoperative cell salvage, postpartum hemorrhage, caesarean section, blood transfusion, pregnancy

## Abstract

**Background:**

Postpartum hemorrhage (PPH) is one of the leading causes of maternal mortality. Promptly recovering blood loss is critical for PPH. Intraoperative cell salvage (ICS) is a method to collect and process red blood cells (RBCs) from the blood lost during surgery and transfuse them to the patient’s circulation during or immediately after surgery. Its effectiveness in reducing the demand for allogeneic blood transfusion has been proven, but its effectiveness and safety as a sole treatment for PPH during Cesarean sections are unclear. This is particularly important for patients who cannot or do not want to accept allogeneic blood transfusion.

**Materials and methods:**

In this prospective randomized controlled study, patients with high risks of PPH were randomized into the ICS group or the control group, receiving ICS or allogeneic RBC transfusion if their hemoglobin level was less than 80 g/L during operation. Data collected include clinical examination, blood cell count, hemoglobin level, coagulation function, and plasma levels of fetal hemoglobin, tissue factor, and alpha-fetoprotein before and after fetal delivery and 0, 2, and 12 h after treatment. Adverse events were recorded.

**Results:**

A total of 130 patients were enrolled, aged 33 ± 1 years with a mean gestation period of 37 ± 1 week. The most common cause of Cesarean section was placenta previa, followed by twin pregnancy, scarred uterus, preeclampsia, placental abruption, fetal distress, and placenta accreta spectrum. Bleeding amount was similar between the two groups. The ICS group, compared to controls, had more efficient increases in levels of hemoglobin, RBC, and hematocrit (all p < 0.05). Coagulation function was maintained in the ICS group but reduced in controls 24 h after transfusion, indicated by significantly reduced fibrinogen level and prolonged prothrombin time (PT), thrombin time (TT), and activated partial thromboplastin time (aPTT) (all p < 0.05). There was a transient but significant decrease in plasma tissue factor and alpha-fetoprotein levels and an increase in plasma fetal hemoglobin level with ICS treatment in the postpartum period. No adverse event occurred with ICS intervention.

**Conclusion:**

ICS is an effective and safe intervention for patients with a high risk of PPH during elective or emergency Cesarean section. It can effectively clear tissue factors and alpha-fetoprotein but not fetal hemoglobin.

## Introduction

Obstetric hemorrhage is a leading cause of maternal mortality, accounting for 27.1% of all maternal deaths (1). The majority of cases of obstetric hemorrhage are postpartum hemorrhage (PPH), which accounts for 2%–3% of all deliveries each year (2) and 25% of total maternal deaths worldwide (3). This figure is even higher in some developing and underdeveloped countries (4, 5). In China, following the implementation of the “three-child policy,” the incidence of PPH due to placenta accreta spectrum, placenta previa, and scarred uterus has increased remarkably, which demands an increase in blood transfusion (6). Although the blood supply is increasing, there is still a big gap between the amount of blood donated and the amount of blood required (7). In addition, allogeneic blood transfusion can cause serious adverse events such as hemolytic transfusion reactions, infections, allergies, immunosuppression, and transfusion-related acute lung injury. Therefore, effective and safe autotransfusion is imperative.

Intraoperative cell salvage (ICS) is a technology that collects red blood cells (RBCs) from the blood lost during surgeries and transfuses them back to the patient’s circulation during or immediately after surgery. ICS is a relatively simple technology, which can effectively and significantly reduce the demand for allogeneic blood transfusions and secondary complications (8). It has been recommended by National Institute for Health and Care Excellent (NICE) guideline for operations where very high volumes of blood loss are expected, including cardiac and complex vascular surgery and major obstetric procedures (9). However, concerns remain in the application of ICS in women undergoing Cesarean sections, mainly the fear surrounding the contamination of amniotic fluid and fetal components in collected blood during the operation (10). In addition, bradykinin-mediated hypotension may occur due to the saturation of ICS during use following a slow reinfusion rate of filter (11). Although modern technology can remove most amniotic fluid and fetal white blood cell components through the washing process and leukocyte-removing filters (11, 12), it cannot distinguish fetal RBCs from maternal RBCs. As a result, fetal RBCs will be collected and returned to the maternal blood, increasing the risk of feto-maternal hemorrhage and erythroblastosis in subsequent pregnancies (13). Currently, the use of ICS for Cesarean sections is still debatable (14). More clinical evidence about its effectiveness and safety is needed, especially in patients with severe obstetric hemorrhage such as PPH.

We therefore conducted a prospective randomized controlled study in patients undergoing Cesarean section with high risks of PPH to determine whether the routine use of ICS during Cesarean section can effectively and safely improve blood loss using blood cell count, coagulation function, and complications following ICS treatment in comparison with standard allogeneic RBC transfusion. In addition, we sought to assess the impact of ICS on risk factors and fetal components that are involved in maternal hemorrhage, including plasma tissue factor (TF), alpha-fetoprotein (AFP), endothelin-1 (ET-1), and fetal hemoglobin.

## Materials and methods

### Study design

**Figure 1:**
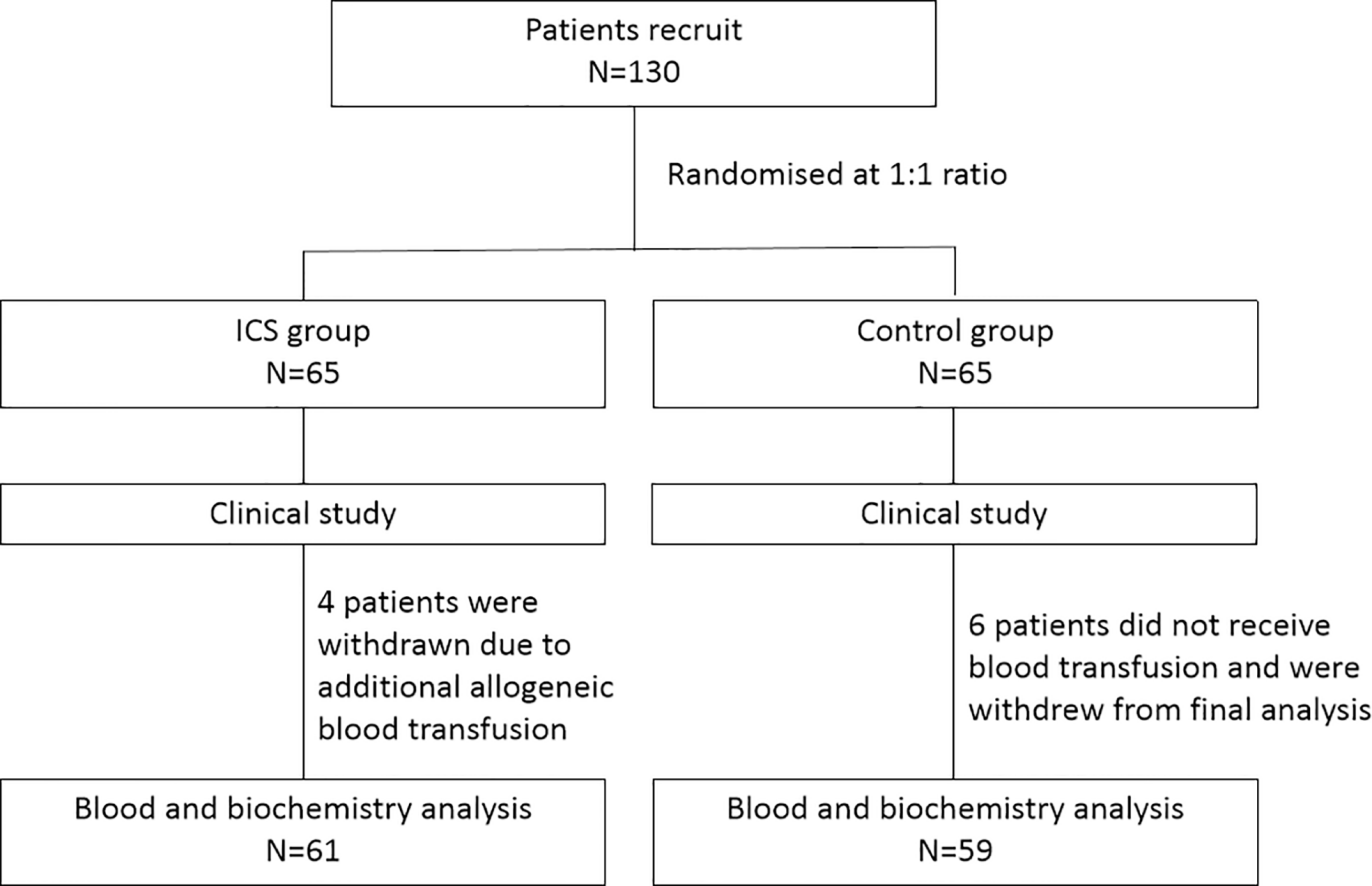
Flow diagram of the study.

This is a prospective randomized controlled study. During the period from August 2017 to December 2019, patients undergoing Cesarean section were recruited and randomized at a 1:1 ratio into either the ICS group or the control group receiving allogeneic RBC transfusion. The study was performed in accordance with relevant guidelines/regulations, and the study protocol was approved by the Ethics Committee of Haidian Maternal and Child Health Hospital, Beijing, China. All participants gave written consents. The study design is shown in **Figure 1**.

Inclusion criteria: 1) 21–45 years old; 2) undergo elective or emergency Cesarean section; 3) have high risks of PPH such as placenta previa and placenta accreta spectrum; 4) history of multiple Cesarean section and PPH.

Exclusion criteria: 1) preoperative hemoglobin (Hb) <90 g/L; 2) comorbidities including cardiovascular disease, hematological diseases, autoimmune disorders, or acute fatty liver/Hemolysis, Elevated Liver enzymes and Low Platelets (HELLP) syndrome; 3) Rh-negative; 4) receiving preoperative anticoagulation therapy or allogeneic RBC transfusion within 3 months before the study; 5) participating in other clinical trials during pregnancy; 6) having any diseases that could lead to the inability to cooperate in the study, such as mental illness and language comprehension disorders; 7) refusing to accept allogeneic RBC transfusion.

Preeclampsia is defined as new onset of hypertension (systolic blood pressure ≥140 mmHg and/or diastolic blood pressure ≥90) accompanied by proteinuria. Placenta previa is defined as the placenta partially or completely covering the cervix. Placenta accreta spectrum is defined as when the placenta grows too deeply into the uterine wall and remains attached partially or completely after delivery. PPH is defined as greater than 1,000 ml estimated blood loss during Cesarean delivery.

### Interventions

All patients underwent Cesarean sections under general or spinal anesthesia. During the operation, ICS or allogeneic RBCs were given to the patients who had a hemoglobin level <80 g/L to achieve a hemoglobin level ≥80g/L at the end of surgery. The transfusion procedure followed the Annex III of the “Clinical Blood Transfusion Technical Guidelines for Surgery and Trauma Patients” of the Ministry of Health, China. The amount of transfusion was recorded.

**Figure 2:**
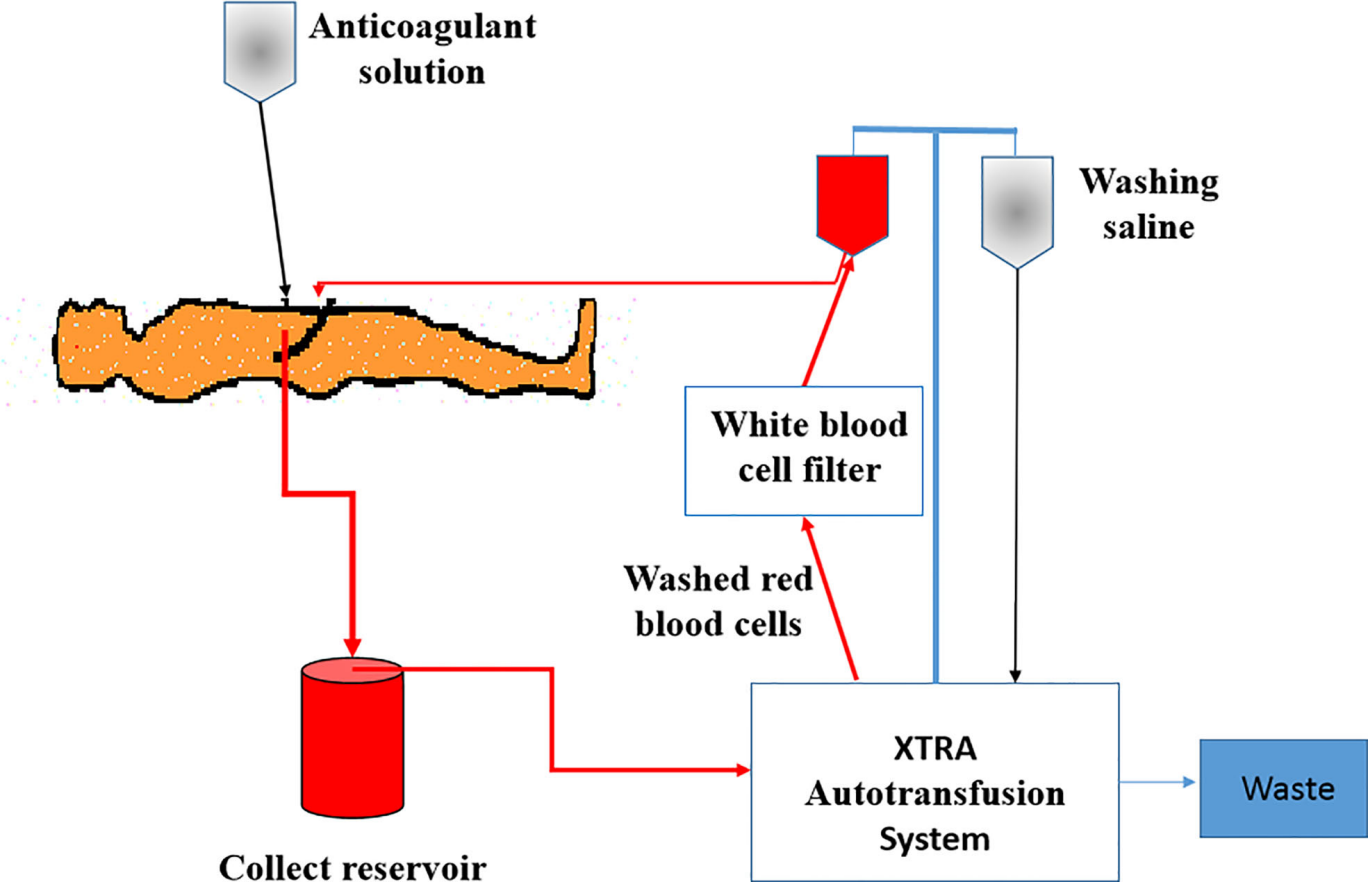
Illustration of intraoperative cell salvage system.

For patients in the ICS group, an XTRA Autotransfusion System (LivaNova, UK) was installed before the operation (**Figure 2**). After delivery and placental separation, an anticoagulant solution (25,000 IU of heparin per 1,000 ml of 0.9% NaCl) was dripped into the operation field and mixed with the maternal blood. Then, the blood mixed with anticoagulant saline solution was sucked into the collection reservoir and centrifuged to separate RBCs from other components in the XTRA Autotransfusion System. RBCs were then washed and suspended with 0.9% saline, passed through a white blood cell filter, and infused back into the patient’s own circulation as soon as possible. If the patient’s hemoglobin was still less than 80 g/L, allogeneic RBCs were provided to increase the hemoglobin level to 80 g/L. Patients in the control group were transfused with allogeneic RBCs. When necessary, plasma and platelet transfusions were given to the patients with severe bleeding in both groups.

### Clinical examination and data collection

Before surgery, all patients underwent baseline assessment. Data collected include age, body weight, and medical history of pregnancy and Cesarean section, weeks of gestation, comorbidities, and placental position. Blood was sampled to evaluate blood cell count, hemoglobin and hematocrit levels, blood gas analysis, electrolytes, liver and kidney function, and coagulation tests including the prothrombin time (PT), activated partial thromboplastin time (aPTT), and thrombin time (TT).

Intraoperative monitoring included vital signs, bleeding volume, hemoglobin level, transfusion volume of allogeneic RBCs, the volume of recollected and transfused ICS, infusion volume of other blood products, and adverse events. The calculation method of intraoperative blood loss: total bleeding amount = blood wet area of gauze (10 cm * 10 cm = 10 ml) + blood volume in the suction bottle, where the blood volume in the suction bottle = the total amount of liquid in the suction bottle - the total amount of flushing anticoagulant solution.

In the ICS group, plasma levels of fetal hemoglobin, TF, AFP, and ET-1 were measured before and after fetal delivery and 0, 2, and 12 h after ICS treatment. In the control group, plasma levels of fetal hemoglobin, TF, AFP, and ET-1 were measured before and after fetal delivery.

The newborns were assessed using the Apgar score system, including heart rate, muscle tone, and other signs at 1 and 5 min after birth to determine if any extra medical care or emergency care is required. A score of 8–10 points is normal, 4–7 points indicate mild asphyxia, and 0–3 points indicate severe asphyxia.

Postoperative monitoring included vital signs, surgical complications, and infections. The following tests were repeated at 24 h and 3 days after Cesarean section, including hemoglobin level, complete blood cell count, hematocrit level, blood gas analysis, electrolytes, liver and kidney function, and coagulation tests.

Biochemistry tests were performed, including chemiluminescence microfluidic immunoassay for AFP, enzyme-linked immunosorbent assay for TF and ET-1, and high-pressure liquid chromatography for fetal hemoglobin.

### Statistical analysis

Statistical analysis was conducted using SPSS version 21.0 (SPSS Inc., Chicago, IL, USA). Quantitative variables are presented as mean ± standard deviation (SD) for data with normal distribution or median (range) for data with non-normal distribution. Comparison was conducted using Student t-test or Mann–Whitney U test. Qualitative variables are presented as number (%) and compared with chi-square test. One-way ANOVA followed by Bonferroni *post-hoc* tests was used to compare the results of the same test at different time points. In this study, p values <0.05 are considered statistically significant.

## Results

During the period from August 2017 to December 2019, 130 patients undergoing Cesarean section were recruited and randomized into either the ICS group (n = 65) or the control group receiving allogeneic RBC transfusion (n = 65) (**Figure 1**). Their baseline clinical characteristics were summarized in **Table 1**. The mean age was 33 ± 1 year, and gestation period was 37 ± 1 week. The most common cause of Cesarean section was placenta previa, accounting for 46.15% in the ICS group and 38.46% in the control group, followed by twin pregnancy, scarred uterus, preeclampsia, placental abruption, fetal distress, and placenta accreta spectrum. Comparing the two groups, no difference was found in terms of patients’ age, body weight, gestation period, causes of Cesarean section, duration of operation, bleeding amount, and Apgar score after 1 and 5 min (all p > 0.05).

**Table 1:** Clinical characteristics of patients.

	ICS group N = 65	Control group N = 65	P value
Age, years	33 ± 1	32 ± 1	0.153
Body weight, kg	71.45 ± 1.30	74.33 ± 1.19	0.175
Gestation period, weeks	37 ± 1	37 ± 1	1.000
Cesarean history, n (%)	17(26.15)	23 (35.38)	0.342
Gestational diabetes, n (%)	16 (24.62)	17 (26.15)	1.000
Causes of Cesarean section, n (%)			
Placenta previa	30 (46.15)	25 (38.46)	0.478
Placenta accreta spectrum	2 (3.08)	2 (3.08)	1.000
Placental abruption	7 (10.77)	9 (13.85)	0.790
Scarred uterus	13 (20.00)	17 (26.15)	0.533
Twin pregnancy	19 (29.23)	14 (21.54)	0.420
Fetal distress	4 (6.15)	4 (6.15)	1.000
Preeclampsia	8 (12.31)	10 (15.38)	0.800
Emergency Cesarean section, n (%)	13 (20.00)	27 (41.54)	0.008
Duration of the operation, min	84.05 ± 5.43	74.92 ± 4.54	0.200
Bleeding amount, ml	971.54 ± 44.83	1084.80 ± 52.97	0.105
Apgar score after 1 min	9.87 ± 0.06	9.56 ± 0.18	0.149
Apgar score after 5 min	10.00 ± 0.00	9.98 ± 0.22	0.943

ICS, intraoperative cell salvage.

In the ICS group, the mean ICS transfusion was 508.92 ± 26.83 ml, and four patients (6.15%) required additional allogeneic RBCs and plasma transfusion due to severe PPH (bleeding amount >2,000 ml). In the control group, the mean allogeneic RBC transfusion was 689.23 ± 50.97 ml; 23 patients (35.38%) had additional plasma transfusion (p < 0.0001 compared with 6.15% in the ICS group); and six patients had no blood transfusion due to small amount of bleeding (<500 ml). Very few patients had platelet transfusion in both groups (one in each group, p = not significant).

In order to exclude confounding effects of allogeneic RBC transfusion, four patients with additional allogeneic RBC transfusion in the ICS group were excluded. Six patients without blood transfusion in the control group were also excluded. Consequently, blood cell count and biochemistry data were collected from 120 patients (61 in the ICS group and 59 in the control group) for further analysis. As shown in **Table 2**, blood cell count, hemoglobin level, and hematocrit were significantly higher in the ICS group than those in the control group following treatment (all p < 0.05). Although hemoglobin level increased to over 100 g/L in all patients after transfusion, it was still significantly lower at the 24-h and 72-h time points than preoperative levels in both groups (all p < 0.05). Blood cell count and hematocrit level were also significantly lower than their preoperative levels in both groups (all p < 0.05). Coagulation function was maintained in the ICS group but reduced in the control group 24 h after transfusion, indicated by significantly reduced fibrinogen level and prolonged PT, TT, and aPTT (all p < 0.05 compared to preoperative levels and those in the ICS group), which returned back to preoperative levels on day 3 after the operation. No significant difference was found in blood gas analysis, electrolytes, and liver and kidney function tests between the two groups and comparing among different time points in the same group (**Table 3**).

**Table 2:** Comparison of perioperative blood count and coagulation function between the two groups.

	ICS group N = 61	Control group N = 59	P value
Hemoglobin, g/L			
Preoperative	115.37 ± 1.90	113.53 ± 1.50	0.318
24 h postoperative	108.16 ± 2.29***	102.80 ± 1.88***	0.016
72 h postoperative	111.90 ± 1.72*	107.76 ± 1.79*	0.046
Red blood cell count, 10^12^/L			
Preoperative	3.72 ± 0.43	3.69 ± 0.42	0.676
24 h postoperative	3.51 ± 0.66**	3.37 ± 0.40***	0.188
72 h postoperative	3.56 ± 0.44*	3.45 ± 0.43**	0.174
Hematocrit, %			
Preoperative	34.27 ± 1.16	33.61 ± 0.39	0.343
24 h postoperative	31.48 ± 1.60***	30.70 ± 0.88***	0.270
72 h postoperative	32.77 ± 1.07*	31.61 ± 1.04*	0.067
Platelet count, 10^3^/μl			
Preoperative	190.89 ± 63.31	183.68 ± 55.62	0.509
24 h postoperative	155.10 ± 34.54***	135.93 ± 36.99***	0.004
72 h postoperative	198.21 ± 63.72	171.32 ± 52.12*	0.002
Plasma fibrinogen, g/L			
Preoperative	4.24 ± 0.55	4.08 ± 0.55	0.099
24 h postoperative	4.09 ± 0.51	3.88 ± 0.52*	0.022
72 h postoperative	4.08 ± 0.58	3.95 ± 0.55	0.238
PT, second			
Preoperative	10.52 ± 0.46	10.55 ± 0.68	0.772
24 h postoperative	10.69 ± 0.54	11.30 ± 1.69*	0.010
72 h postoperative	10.47 ± 0.56	10.31 ± 2.36	0.614
aPTT, second			
Preoperative	26.63 ± 2.54	26.39 ± 3.12	0.642
24 h postoperative	26.89 ± 2.47	28.63 ± 2.41*	0.0001
72 h postoperative	26.92 ± 2.04	27.47 ± 2.38	0.175
TT, second			
Preoperative	13.27 ± 1.68	13.80 ± 1.74	0.091
24 h postoperative	13.66 ± 0.69	14.97 ± 2.26*	0.0001
72 h postoperative	13.82 ± 0.87	13.67 ± 1.74	0.544

ICS, intraoperative cell salvage; PT, prothrombin time; aPTT, activated partial thromboplastin time; TT, thrombin time.

*p < 0.05, **p < 0.01, ***p < 0.0001 compared to the preoperative level.

**Table 3:** Comparison of perioperative gas analysis, electrolytes, and liver and kidney function between the two groups.

	ICS group N = 61	Control group N = 59	P value
PaCO_2_, mmHg			
Preoperative	35.04 ± 0.36	35.66 ± 0.37	0.910
24 h postoperative	35.13 ± 0.35	35.63 ± 0.42	0.859
72 h postoperative	35.39 ± 0.33	35.42 ± 0.29	0.955
HCO_3_, mmol/L			
Preoperative	23.15 ± 0.21	23.39 ± 0.24	0.676
24 h postoperative	22.08 ± 0.16	21.46 ± 0.13	0.093
72 h postoperative	23.19 ± 0.19	23.47 ± 0.22	0.563
Ca2+, mmol/L			
Preoperative	1.12 ± 0.01	1.12 ± 0.01	0.920
24 h postoperative	1.13 ± 0.01	1.11 ± 0.01	0.203
72 h postoperative	1.12 ± 0.01	1.12 ± 0.01	0.910
K+, mmol/L			
Preoperative	3.73 ± 0.04	3.74 ± 0.03	0.847
24 h postoperative	3.62 ± 0.40	3.70 ± 0.33	0.080
72 h postoperative	3.74 ± 0.04	3.76 ± 0.04	0.774
ALT, U/L			
Preoperative	15.78 ± 0.94	14.91 ± 0.64	0.909
24 h postoperative	16.33 ± 0.93	15.03 ± 0.72	0.281
72 h postoperative	16.06 ± 0.78	15.11 ± 0.59	0.336
AST, U/L			
Preoperative	16.74 ± 0.53	17.91 ± 0.62	0.153
24 h postoperative	18.35 ± 0.48	19.53 ± 0.55	0.108
72 h postoperative	17.70 ± 0.74	18.90 ± 0.71	0.242
Creatinine, µmol/L			
Preoperative	46.01 ± 0.85	47.36 ± 1.03	0.312
24 h postoperative	48.03 ± 0.98	50.11 ± 1.10	0.160
72 h postoperative	48.41 ± 0.73	48.88 ± 1.15	0.729
Urea, mmol/L			
Preoperative	13.27 ± 1.68	13.80 ± 1.74	0.412
24 h postoperative	13.66 ± 0.69	14.97 ± 2.26	0.315
72 h postoperative	13.14 ± 0.09	13.29 ± 0.08	0.544

ICS, intraoperative cell salvage; ALT, alanine aminotransferase; AST, aspartate transaminase.

**Figure 3:**
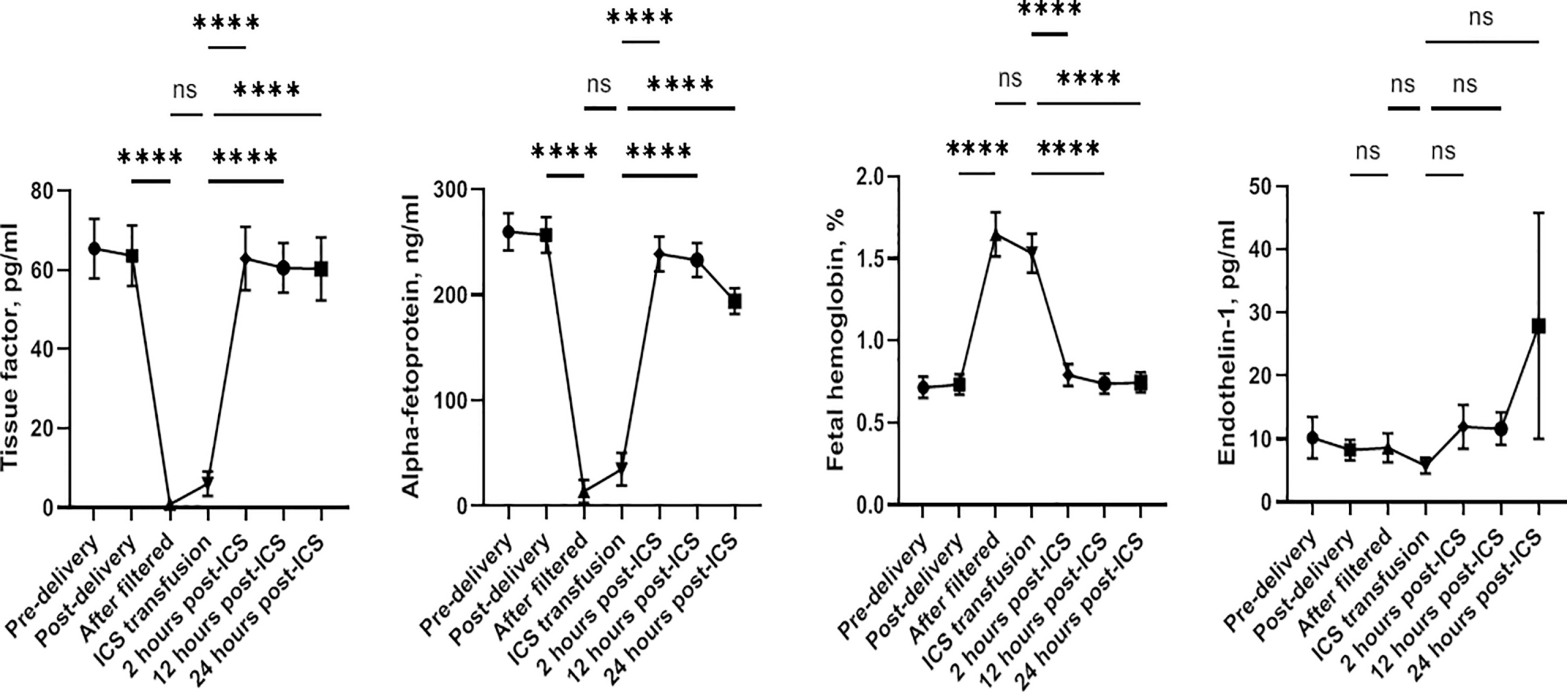
Changes of plasma levels of tissue factor, alpha-fetoprotein, fetal hemoglobin, and endothelin-1 before and after delivery and after blood transfusion. ****p < 0.0001; ns, not significant.

Plasma levels of TF, AFP, fetal hemoglobin, and ET-1 were not significantly different before and immediately after child delivery in both groups (all p > 0.05). Furthermore, no difference was found in any of these factors between the two groups (all p > 0.05) (**Table 4**). To further investigate the impact of the ICS process and transfusion on TF, AFP, fetal hemoglobin, and ET-1 levels, we examined blood samples before and after delivery and 0, 2, 12, and 24 h after transfusion in the ICS group. Their levels in RBC suspension after filtration were also measured. As shown in **Figure 3**, TF and AFP levels were significantly lower in RBC suspension after filtration, indicating that these two factors were significantly filtered by the ICS process. Their levels were continuously low in maternal blood immediately after the ICS transfusion but returned to pretransfusion levels after 2 h following transfusion. In contrast, fetal hemoglobin level significantly increased in the filtered blood sample and was continuously high in maternal blood after ICS transfusion but reduced to pretransfusion level after 2 h. Plasma ET-1 level was not significantly affected by the ICS process and transfusion. Moreover, plasma AFP levels before and after delivery were significantly higher than normal range (10–150 ng/ml).

**Table 4:** Comparison of plasma fetal hemoglobin, endothelin-1, tissue factor, and alpha-fetoprotein between the two groups before and after child delivery.

	ICS group N = 61	Control group N = 59	P value
Fetal hemoglobin, g/L			
Preoperative	0.71 ± 0.06	0.70 ± 0.04	0.882
After delivery	0.72 ± 0.06	0.71 ± 0.03	0.767
Endothelin-1, pg/ml			
Preoperative	10.20 ± 3.29	14.66 ± 4.08	0.396
After delivery	8.25 ± 1.60	16.86 ± 6.33	0.192
Tissue factor, pg/ml			
Preoperative	65.42 ± 7.50	75.08 ± 5.27	0.294
After delivery	63.61 ± 7.64	79.63 ± 9.04	0.178
Alpha-fetoprotein, ng/ml			
Preoperative	259.76 ± 17.50	250.37 ± 13.31	0.670
After delivery	256.85 ± 17.09	251.14 ± 13.17	0.792

ICS, intraoperative cell salvage.

Within 2 h of ICS, no patient showed worsening bleeding. No adverse events such as amniotic fluid embolism, infection, hypotension, allergy, adverse cardiovascular events, or delayed PPH were reported in the ICS group. In the control group, two patients had rashes following allogeneic RBC transfusion but recovered after intravenous injection of dexamethasone 10 mg. No other adverse events and complications were reported.

## Discussion

In this prospective randomized clinical trial, we evaluated the effectiveness and safety of ICS implementation in patients with a high risk of PPH who underwent elective or emergency Cesarean sections. Our results indicated that ICS treatment increased hemoglobin level more effectively and maintained coagulation function compared to allogeneic RBC transfusion. Although ICS treatment led to a transient decrease in plasma TF and AFP levels and an increase in plasma fetal hemoglobin level in the postpartum period, such changes only lasted for less than 24 h. There was no adverse effect such as amniotic fluid embolism, infection, hypotension, or allergy following ICS treatment.

PPH is a leading cause of maternal mortality. How to effectively improve blood loss by blood transfusion in PPH patients is therefore critical. Substantial data from previous studies have supported the use of ICS technology in elective and emergency Cesarean sections (15). However, the primary outcome in most trials is to evaluate the rate of autologous RBC transfusion and whether it can reduce allogeneic RBC transfusion (16). Recently, Liu et al. (17) demonstrated that ICS significantly reduced the amount of allogeneic RBC transfusion, the incidence of adverse effects, and hospital stay in patients with severe bleeding. Zeng et al. (18) also indicated that ICS helped to reduce the need for allogeneic RBC and fluid transfusion in patients with placenta accreta spectrum. To our knowledge, the present study is the first randomized controlled trial to evaluate the efficacy and safety of ICS as a monotherapy in patients with a high risk of PPH. Our findings indicate that compared to standard allogeneic RBC transfusion, ICS transfusion can effectively improve the hemoglobin level in patients with predictably high rates of hemorrhage if the ICS system is set up preoperatively. The difference between the two groups can still be detected 3 days after the operation, indicating that ICS can preserve RBCs well and maintain their function. Previously, Salaria et al. has found RBC cell membrane deformability, which persisted beyond 3 postoperative days. It was related to transfusion with stored allogeneic RBCs but not salvaged RBCs in cardiac surgery patients (19). Our findings further confirm the benefit of intraoperatively salvaged RBCs in obstetric patients.

One of our main findings is that coagulation function was deteriorated with allogeneic RBC transfusion but not with ICS treatment. The underlying mechanisms may be related to the significantly reduced platelet count and plasma fibrinogen level in the controls. A previous study by Yang et al. (20) demonstrated that massive allogeneic RBC transfusion could result in a decrease in platelet count, although coagulation function was still comparable. In the present study, although the amount of ICS and allogeneic RBC transfusion was not significantly different, additional plasma administration was provided to 35.38% of patients in the control group. As a result, the expansion of blood volume following plasma transfusion might reduce the platelet count and fibrinogen level, contributing to prolonged PT, aPTT, and TT. In clinical practice, carefully monitoring the coagulation function after blood and plasma transfusion is really necessary.

There are concerns that the slow reinfusion rate of filter may cause the saturation of ICS, thereby inducing bradykinin-mediated hypotension (11). However, no hypotension event was reported in our PPH patients following ICS treatment, indicating the safety of ICS treatment in PPH patients undergoing elective or emergency Cesarean sections. We believe that the preoperative setup of the ICS system can ensure an effective ICS treatment, which helps to reduce or avoid allogeneic RBC transfusion.

Amniotic fluid embolus is a rare but vital condition during child delivery, accounting for high mortality and morbidity rates (21). It is one of the most concerning adverse effects of autologous blood recovery. Therefore, evaluating surrogate markers associated with amniotic fluid embolus will be helpful. TFs play an important role in the mechanisms of amniotic fluid embolus (22). The plasma level of TFs may be useful as an indicator of amniotic fluid embolus. We found that the preoperative level of TF was very low. It further reduced significantly in filtered RBC suspension and in maternal blood immediately after ICS transfusion and then returned to preoperative levels after 2 h. This indicates that TFs can be completely washed out during the process of ICS. Our findings are consistent with those of previous reports (23). We also found that the plasma level of AFP had a similar change pattern as TF, but its levels before and after delivery were significantly higher than normal range. The underlying mechanism is not clear. In a previous study, the elevation of plasma AFP level was supposed to be able to predict pregnancy risk including feto-maternal hemorrhage (24). Further investigation is needed.

In contrast to TF and AFP, fetal hemoglobin contamination in the blood collected during Cesarean sections is impossible to be cleared by the ICS system. We found that the fetal hemoglobin level was significantly elevated in the filtered RBC suspension and in maternal blood immediately after ICS transfusion, suggesting fetal hemoglobin contamination. Although its level decreased after 2 h, the isoimmunization secondary to the exposure to fetal hemoglobin may lead to erythroblastosis in subsequent pregnancies. In contrast to our findings, Sullivan and Ralph (25) have reported that fetal RBCs are present in the maternal circulation throughout pregnancy and that the volumes are comparable to that obtained from ICS. In this study, majority of patients had vaginal delivery and blood loss ranged from 100 to 2,300 ml, which are different from those in the present study. Nevertheless, it is worthwhile to investigate further.

The present study has some limitations. This is a single-center study. The number of participants recruited in each group is only 65. Nevertheless, all patients recruited had risk factors of PPH. Our findings still provide solid evidence for the implementation of ICS in patients with a high risk of PPH.

In conclusion, ICS is an effective and safe intervention for blood loss recovery in patients with a high risk of PPH during elective or emergency Cesarean section. The ICS process can effectively clear TFs and AFP but not fetal hemoglobin. It is necessary to monitor isoimmunization for future pregnancies.

## Data availability statement

The original contributions presented in the study are included in the article/supplementary material. Further inquiries can be directed to the corresponding author.

## Ethics statement

The studies involving human participants were reviewed and approved by Ethics Committee of Haidian Maternal and Child Health Hospital. The patients/participants provided their written informed consent to participate in this study.

## Author contributions

BL and LW contributed to conception and design of the study. BL, MG, XD, SH, XL, and YW involved in data collection. BL performed the statistical analysis and wrote the first draft of the manuscript. All authors contributed to manuscript revision, read, and approved the submitted version. LW was the lead of study and funding holder.

## Funding

This study was supported by the Beijing Science and Technology Commission Research Fund (225,000 yuan). Project number: Z171100001017151.

## Acknowledgments

We would like to thank Prof. Hongmei Jia, Prof. Hongqing Jiang, Prof. Fengqiu Li, Prof. Dongru Cao, Dr. Jinxin Hao and Dr. Juan Zuo for their valuable support.

## Conflict of interest

The authors declare that the research was conducted in the absence of any commercial or financial relationships that could be construed as a potential conflict of interest.

## Publisher’s note

All claims expressed in this article are solely those of the authors and do not necessarily represent those of their affiliated organizations, or those of the publisher, the editors and the reviewers. Any product that may be evaluated in this article, or claim that may be made by its manufacturer, is not guaranteed or endorsed by the publisher.
